# RASolute 302: lessons learned and the road ahead in pancreatic cancer

**DOI:** 10.1093/oncolo/oyag346

**Published:** 2026-08-28

**Authors:** Andrew H Ko

**Affiliations:** Division of Hematology/Oncology, University of California San Francisco, San Francisco, CA 94143, United States

**Keywords:** pancreatic cancer, RAS inhibitor, daraxonrasib, clinical trial, dropout

It was a watershed moment when data from the phase III RASolute 302 trial were publicly released, demonstrating the significant and, in fact, striking, improvement in overall survival for patients with previously treated metastatic pancreatic cancer receiving the multiselective RAS inhibitor daraxonrasib compared to standard second-line chemotherapy.^1^ In a disease where clinical trial success has typically been marked by fairly modest incremental gains, a hazard ratio for death of 0.40 (as reported in RASolute 302) was virtually unprecedented. Particularly heartening was the relatively minimal attenuation in efficacy outcomes in this phase III study when compared to prior phase I/II data^2^—a trend so commonly observed when novel agents advance through stages of clinical testing, especially in this disease—with a median overall survival that remained greater than 13 months in the study cohort. Encouragingly, all other clinically relevant endpoints (progression-free survival, objective response rate, and global quality of life) were significantly and meaningfully in favor of daraxonrasib as well. Meanwhile, the unique side effects associated with this class of drugs, most notably rash, oral stomatitis, and diarrhea, varied in severity and required dose interruption and/or interruption in more than half of patients but were mostly manageable through an aggressive prophylactic and early management supportive care plan.

Aside from its robust efficacy, an additional reason daraxonrasib (and RAS inhibitors in general) have generated such excitement comes down to the drug’s mechanism of action. A deeper understanding of the structure and biology of the RAS protein, which prompted several novel and exquisite approaches to drug development, has resulted in this once“undruggable” molecule—so central to the growth and survival of pancreatic cancer and many other solid tumors–becoming amenable to successful therapeutic targeting, to the proven benefit of patients. Recognizing the urgency and time sensitivity so many patients with pancreatic cancer face, the U.S. Food and Drug Administration quickly authorized an Expanded Access Program (EAP)^3^ so that daraxonrasib could be made accessible to eligible patients prior to its FDA approval; at the time of this writing, this EAP is fully operational, with what appears to be overwhelming demand.

While RASolute 302 represents an unqualified success and important advance in the field, important lessons can be taken from this study that may help guide the conduct of ongoing and future trials of RAS inhibitors. In particular, when one looks at the CONSORT diagram from RASolute 302, 11.5% of patients randomized to the control arm withdrew prior to starting treatment, with an additional 10.3% discontinuing chemotherapy during the study for reasons other than disease progression or toxicity (compared to 0.4% and 3.6%, respectively, for the experimental arm).^1^ Imbalance in withdrawal rates always represents a challenge in non-placebo-controlled randomized clinical trial design, as it may affect the interpretability of study results. Specifically, differential attrition between arms introduces the risk of post-randomization selection bias, sometimes informally referred to as “disappointment bias.”^4^^,^^5^ This concept may have affected enrollment to RASolute302, with patients eager to be randomized to the experimental arm, for which promising early data from the phase I/II trial had already been reported. The FDA’s Breakthrough Therapy designation of daraxronasib^6^ in June 2025, right in the midst of trial enrollment, undoubtedly compounded matters. It is easy to see how the integrity of a trial can risk being compromised due to a confluence of factors that may contribute to early dropout, including: (1) highly promising early results of the agent under investigation—first reported at scientific meetings, then picked up by trade journals and biotech reports, and eventually making their way to general news outlets and social media—that create considerable buzz prior to and during study enrollment; (2) a highly lethal disease where there may be a short window of opportunity to receive any treatment; (3) a less-than-appealing control arm; and (4) a trial design that does not allow crossover. The take-home message: while every effort was appropriately made in RASolute 302 to prevent dropout on the control arm, it still posed a major challenge—and will undoubtedly continue to be an issue that current and future RAS inhibitor trials will need to proactively address, especially as study patients may become increasingly unwilling to be randomized to treatment with chemotherapy alone.

As RAS inhibitors assume an increasingly prominent role in the management of patients with pancreatic cancer, it will be essential for oncologists to avoid the temptation of using these agents beyond their approved indication, until sufficient data dictate otherwise. One can envision treating providers in real-world practice–perhaps with the encouragement or pleading of patients—prescribing daraxonrasib instead of standard chemotherapy in the front-line setting for metastatic disease or combining the two approaches together; as well as using daraxonrasib for earlier-stage disease as adjuvant and/or neoadjuvant therapy. While a number of ongoing trials are exploring RAS inhibitors in these exact clinical contexts,^7–9^ it is still too premature to adopt these as standard clinical practice at the present time. At the very least, there is likely to be a low threshold to pivot away from front-line chemotherapy sooner than one might otherwise, for example if a patient is experiencing bothersome (but still manageable) side effects from chemotherapy or has achieved reasonable disease control but not a dramatic response, with the enticement of making an early switch to a RAS inhibitor. Additionally, the drug will undoubtedly be offered to a broader range of patients, including those with poorer performance status than those enrolled on RASolute 302 (which was limited to individuals with ECOG PS 0-1), as well as with marginal renal or hepatic function or suboptimal nutritional status. Data for these higher-risk subgroups are currently lacking, highlighting the importance of both prospective trials focusing on these more vulnerable patients as well as publication of real-world data to inform our approach. Whether these (or other) patients are at higher risk for severe adverse events–such as refractory cutaneous toxicity that requires dermatologic expertise–and/or would benefit from a lower starting dose of medication, needs to be better understood. Availability of onco-dermatologists, a sub-specialty which presently remains primarily in the purview of academic medical centers, will be an increasingly essential component of cancer care to allow more patients to achieve maximal benefit by maintaining optimal dose intensity while preserving quality of life.

Another critical point worth noting here: while the benefit of daraxonrasib appeared to be retained in patients whose tumors harbored RAS mutations outside of codon 12, and even those without a detectable RAS mutation, these “other” patients comprised only a small proportion (less than 10 percent) of the RASolute-302 study population. This underscores the vital importance of gathering more data on the efficacy of RAS inhibitors specific to these molecular subgroups. Along these same lines, even if daraxonrasib receives FDA approval irrespective of RAS mutational status, it remains essential for oncology providers to continue ordering comprehensive genomic profiling on all patients, ideally via interrogation of tumor tissue or, if need be, by circulating tumor (ct)DNA. This will be useful not only for picking up other potentially actionable genetic alterations, but also for identifying the specific KRAS genotype for any given patient. Such information may become increasingly relevant if allele-specific RAS inhibitors (eg, those that target G12D)—which may have certain advantages over “pan-RAS” inhibitors, in particular exhibiting a more favorable side effect profile^10^^,^^11^—ultimately find their way into clinical practice.

One of the next key frontiers we will be facing in the clinical management of patients with pancreatic cancer will be determining the most effective treatment strategies once resistance to RAS inhibitors develops. We are still awaiting data from RASolute 302 regarding how patients fare with salvage therapies, including a return to standard chemotherapy, in this setting. Re-biopsying the tumor at the time of disease progression is always desirable to try to elucidate mechanisms of resistance but is easier said than done in clinical practice, while ctDNA may or may not identify emergent mutations. Available data suggests a number of different mechanisms of resistance, both genetic and non-genetic, to patients who have been treated with daraxonrasib, at least some of which may potentially be overcome with alternative targeted therapies.^12–15^ In addition to the importance of real-world data to inform post-progression prognosis and response to salvage therapies following exposure to RAS inhibitors, future clinical trials will need to evaluate novel strategies tailored specifically to the altered tumor biology that arises in this context.

The demonstrable benefit of RAS inhibitors in pancreatic cancer represents arguably the most important therapeutic advance we have had to date for this disease, following decades of modest incremental gains or, more commonly, negative trial results. While justifiably offering a great deal of optimism and excitement for patients, providers, and researchers alike, it is clear that much more work needs to be done. In summary, RASolute 302 and daraxonrasib represent a landmark achievement, but hopefully only mark the beginning of a transformative way in which we treat and improve the longevity and quality of life of our patients with pancreatic cancer.
